# Left Atrial Myxoma in Pregnancy: Management Strategy Using Minimally Invasive Surgical Approach

**DOI:** 10.1155/2017/8510160

**Published:** 2017-04-16

**Authors:** Noppon Taksaudom, Kuntharee Traisrisilp, Rungsrit Kanjanavanit

**Affiliations:** ^1^Cardiovascular and Thoracic Surgery Unit, Department of Surgery, Maharaj Nakorn Chiang Mai Hospital, Chiang Mai University, Chiang Mai, Thailand; ^2^Department of Obstetrics and Gynecology, Maharaj Nakorn Chiang Mai Hospital, Chiang Mai University, Chiang Mai, Thailand; ^3^Cardiology Unit, Department of Internal Medicine, Maharaj Nakorn Chiang Mai Hospital, Chiang Mai University, Chiang Mai, Thailand

## Abstract

This case report concerns a young woman who, during her pregnancy, suffered severe mitral regurgitation. It was discovered at the same time that she had a left atrial myxoma. During the early postpartum period she successfully underwent an anterior minithoracotomy to remove the left atrial myxoma in conjunction with repair of the mitral valve. The thoracotomy approach in this specific patient was chosen as it would give a better chance of successful mother-child bonding because the patient would be able to avoid the precautions which would have been necessary following a sternotomy, especially the limitation of her ability to hold her child during the first 4–6 weeks postoperatively.

## 1. Introduction

Cardiac myxoma is the most common type of benign cardiac tumor which can occur at any age. It occurs mostly in women and is sometimes detected during pregnancy [1]. Cardiac myxoma in pregnant woman is extremely rare; the most recent literature review published in 2015 reported only 51 cases from 44 publications, and its management is also very difficult [2]. The overall perinatal mortality is very high in pregnant woman undergoing cardiac surgery especially if the cardiopulmonary bypass (CPB) is used and in early gestational period [3]. The management decision in this particular case drew on the experience of numerous specialists including a cardiologist, cardiac surgeon, obstetrician, pediatrician, and a neonatologist. The multidisciplinary team approach has a crucial role in ensuring the best outcome for both mother and child [4]. The standard operative technique for dealing with a cardiac myxoma is the median sternotomy approach which allows the removal of the mass under cardiopulmonary bypass [1]. However, the sternal precautions needing to be applied after the sternotomy in order to avoid sternal dehiscence include using wound support, lifting restrictions, mobility aid restrictions, and driving restrictions [5]. These restriction protocols, especially weight lifting restrictions, may lead to poor child-mother bonding especially during the first 3 to 6 months after surgery and may interfere with breast feeding. In this particular complex situation, the operation was not solely dependent on the surgeon's preference alone but a holistic approach from both the family's perspective and the medical team needed to be applied.

This report describes the case of an immediate postpartum patient with a very large left atrial myxoma and severe mitral regurgitation. The corrective surgery was carried out via the minithoracotomy approach in Chiang Mai University Hospital.

## 2. Case Report

**Figure 1:**
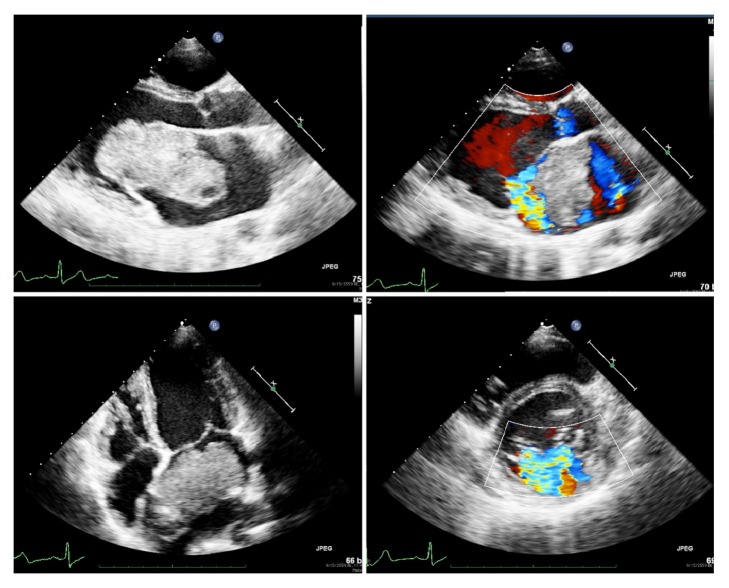
Preoperative echocardiography showed huge left atrial mass protruding through mitral valve with severe mitral insufficiency.

A 28-year-old Burmese pregnant woman was referred to our institute because of complicated pregnancy. Two years previously, she had a history of progressive dyspnea and orthopnea. A thorough investigation was carried out, a large left atrial mass was diagnosed, and she was scheduled for surgery. Due to economic problems and poor communication the patient was lost from the schedule and all attempts to contact her failed. She arrived at the hospital again and was pregnant. The pregnancy was unplanned and she had had no antenatal care. The estimated gestation by ultrasound was 24 weeks. The patient reported constant dyspnea on exertion in NYHA II without any progression in symptoms. On physical examination, vital signs were stable with a pansystolic murmur grade IV at apex. The chest film showed cardiomegaly and the cardio : thoracic ratio was 60%. Electrocardiography showed a normal sinus rhythm with left ventricular hypertrophy by voltage criteria. On echocardiographic examination, as shown in Figure 1, a large, smooth surface, mobile mass, measuring at least 9 × 4 cm was found in the left atrium. There was no calcification or bleeding in the mass. The stalk of the mass was attached to the interatrial septum. The mass protruded into the mitral valve orifice causing both significant left ventricular inflow obstruction and severe mitral regurgitation with mitral annular dilation. The left ventricle was dilated with a good ejection fraction at 70.1%. Left ventricular systolic and diastolic volumes were 53 mL and 157 mL, respectively. The estimated pulmonary pressure was 75 mmHg which indicated severe pulmonary hypertension.

The patient was admitted immediately. A multidisciplinary team was established to deal with this special situation including cardiac surgeons, cardiologists, obstetricians, anesthesiologists, pediatrics, and neonatologists. After extensive discussion, the conclusion was to wait for lung maturity and schedule her for an epidural, painless vaginal delivery with a back-up emergency plan for worst case scenario. At the 32nd week of gestation, corticosteroids were given to promote pulmonary maturity and labor was induced. Intensive fetal and maternal monitoring occurred at every step. A painless vaginal delivery was enabled by epidural anesthesia and only a mild degree of pulmonary edema was detected during labor. The male preterm neonate was considerably healthy for his age, with a weight of 1,900 gm and APGAR scores of 7 and 9 at 1 and 5 minutes. The patient was returned to the cardiac intensive care unit for intensive monitoring. There was no clinical deterioration and she was still in NYHA II.

**Figure 2:**
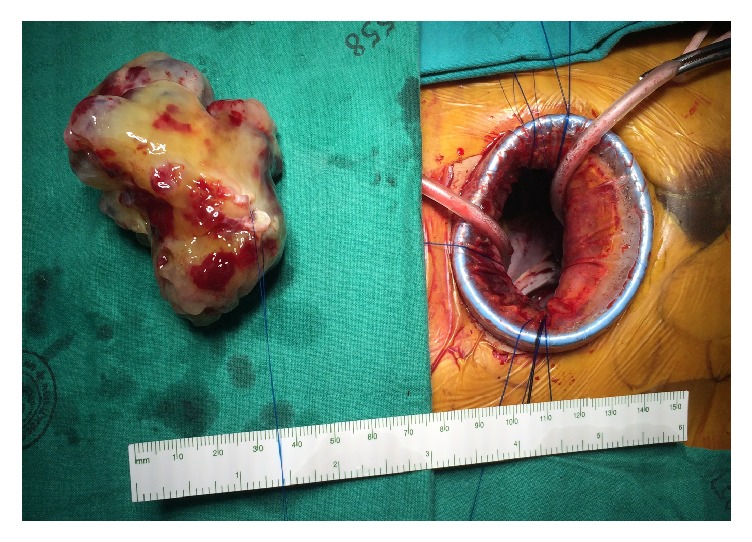
The large mass after removal compared to the incision.

Two weeks after delivery, the patient was scheduled for cardiac surgical correction. All preoperative investigations were within normal limits. The operative procedure was conducted under general anesthesia with single-lumen intubation. A central venous catheter was placed on the left internal jugular vein and a 6 Fr vascular sheath was placed on the right internal jugular vein. Transesophageal echocardiography was set up routinely. The patient was placed in a supine position with an inflatable bag placed beneath her right chest wall elevating the right side of the chest to achieve greater exposure. Standard antiseptic preparation was made. A 3 cm right inguinal incision was made allowing exploration of the common femoral artery and vein. Purse string sutures were placed on both vessels. A right submammary 5 cm incision was made and the pleural cavity was entered via the 4th intercostal space. Skin protector was placed round the incision without using a rib spreader. The patient was full heparinized and peripheral CPB was established from the right groin. The femoral arterial and venous cannulas were size 17 Fr and 20 Fr, respectively. Both cannulations were performed using Seldinger's technique under TEE guidance. An additional venous cannula, number 14 Fr, was placed percutaneously via the right internal jugular vein again using Seldinger's technique. A thoracoscopic port was inserted via the 3rd intercostal space at the midaxillary line. After achieving full bypass, ventilation was stopped and the lungs were collapsed. The pericardium was opened longitudinally from the superior vena cava (SVC) to the inferior vena cava (IVC) and 2 to 3 cm anterior to the right phrenic nerve. Both SVC and IVC were snared with large heavy silk. A cardioplegic cannula was placed in the ascending aorta. A Chitwood aortic clamp was passed from a separate small incision through the 2rd intercostal space at the midclavicular line. An aortic cross clamp was applied and the heart was arrested by the antegrade route with crystalloid Histidine-Tryptophan-Ketoglutarate solution. Both SVC and IVC were fastened to achieve total bypass. The right atrium was opened along the atrioventricular groove. The interatrial septum was incised at the lower border of the fossa ovalis. A hanging stitch was placed and the interatrial septum was excised keeping 0.5 to 1 cm from the stalk of tumor. The tumor was removed easily without any adhesion with the residual atrial wall or mitral valve. The mass was delivered, with some additional rib traction, via submammary thoracotomy incision. The reddish bulky tumor was 8 cm × 5 cm × 6 cm in size and had a smooth and sleek surface with a small dense stalk attached to the limbus of the fossa ovalis as shown in Figure 2. The left ventricular venting cannula was placed after the mass was removed. Mitral valve analysis was done and revealed a grossly normal valve apart from a significant annular dilation. A saline test also showed a significant regurgitation. A mitral valve annuloplasty was done with Carpentier-Edwards Physio annuloplasty ring (Edwards Lifesciences, Irvine, CA, USA) number 34. The atrial septal defect was closed directly using continuous polypropylene 4/0 running sutures. The right atrial wall was closed. After rewarming and deairing, the aortic cross clamp was removed and the CPB was weaned off and terminated uneventfully. All wounds were closed and one chest drain was placed in the right pleural cavity. Total operative time was 3 hours and 40 minutes; cross clamp time and total bypass time were 105 and 160 minutes, respectively. Intraoperative TEE showed good results; there was no residual atrial defect and a resultant good competent mitral valve. The patient was extubated the next morning 14 hours after the operation; then she had a normal recovery with no significant complications. The chest drain was removed on the 2nd postoperative day and the chest film was normal.

**Figure 3:**
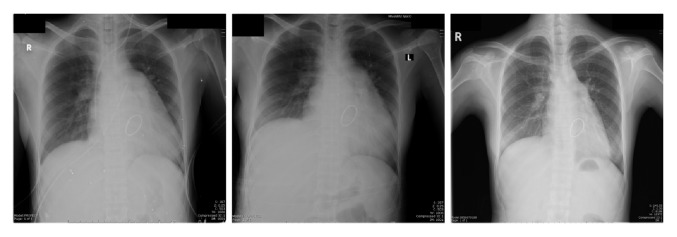
Serial of postoperative chest X-rays showed gradual reduction in cardiac size.

The patient was discharged 7 days after surgery. There was a delay of 3 days due to her preterm baby's condition. She was clinically stable in NYHA I. However, early postoperative echocardiography showed severe reduction in ejection fraction of 20% with competent valvular function and no residual tumor. Left ventricular systolic and diastolic volumes were 102 mL and 131 mL, respectively. However, heart size from the chest film gradually decreased on each visit until it became normal size as demonstrated in Figure 3. She was prescribed heart failure medications and warfarin as the mother could not breast feed due to no lactation occurring. Serial echocardiography 2 months postoperatively showed cardiac function was significantly improved with an ejection fraction of 42% and normal left ventricular size (left ventricular systolic and diastolic volumes were 38 mL and 72 mL, resp.). Pathology report later confirmed the diagnosis of myxoma.

## 3. Discussion

Primary tumors of the heart are relatively rare and the most common are atrial myxomas which account for 50% of all primary cardiac tumors [1]. Left atrial myxoma in pregnant patients is extremely rare, only 51 cases being reported in the most recent literature review in 2015 [2]. From the comprehensive review the management of tumors in pregnancy is very varied; procedures include termination of pregnancy, tumor resection during pregnancy, delay in resection until the 3rd trimester is reached, or delayed cardiac surgery until after delivery. Both maternal and fetoneonatal outcomes are dependent on the shorter gestational age and the use of CPB [3, 6]. A previous publication suggested attempted delivery ahead of surgery/CPB or to defer surgery till late pregnancy [3]. However, cardiac myxoma has the associated risk of embolism especially in the hypercoagulable condition of pregnancy. Therefore, another publication suggested that a surgical excision be performed in all pregnant women [4]. In our case, this was a 28-year-old pregnant patient with a long history of left atrial myxoma without any history of embolism and she had both a very large left atrial myxoma and a significant mitral valve insufficiency which may have necessitated a more complicated operation and longer duration of surgery. From these reasons, our multidisciplinary team, including a cardiologist, cardiac surgeon, obstetrician, pediatrician, and neonatologist, reached a consensus in the management of this patient to delay the operation until 32 weeks' gestation was reached increasing neonatal lung maturity. This patient successfully delivered, well-being being maintained using intensive monitoring of both fetal and maternal parameters without any significant complications.

The median sternotomy was considered as a standard surgical approach for myxoma removal with a very low complication rate. However, there were numerous concerns about sternal precautions needed after the sternotomy to prevent sternal instability especially regarding weight lifting restrictions. There was no exact weight specified although data from a web-based survey in Australia by Tuyl et al. reported the common use of a weight restriction of 2 to 5 kg [5]. This may interfere with a normal maternal-child relationship and bonding because the mother would need to avoid holding her child due to pain and breast feeding may be difficult. In this case, we selected a right anterior minithoracotomy approach in order to avoid these issues.

Several publications have reported using a minimally invasive approach in the treatment of cardiac myxoma [7–9]. Yang et al. also reported higher quality of life using a robotically assisted approach in atrial myxoma excision [10]. An anterior thoracotomy approach however is an excellent approach via both the right and left atria including the advantage of good mitral valve exposure. In our institute, minimally invasive mitral valve surgery is one of the standard approaches for simple valvular pathology and this has enhanced our experience in the minimally invasive field so we were not reluctant to use the minimally invasive approach for complex myxoma removal and mitral valve surgery. To enable a smaller incision, a video-assisted approach is a crucial adjunct procedure because the mitral valve visualization could be very difficult if the incision is placed medially to the right atrium. The patient had a rapid recovery and could have been discharged on the 4th day postoperatively but she needed to be admitted until her child was discharged. The poor ejection fraction recorded on the echocardiogram after surgery may represent the real ventricular function resulting from the eradication of the regurgitation through mitral insufficiency. However, the poor myocardial protection during minimally invasive approach is also considered as one cause of this myocardial suppression. In this case, we used single dose of crystalloid Histidine-Tryptophan-Ketoglutarate solution. This cardioplegic solution needs proper topical cooling in the pericardial sac during operation. But from minimally invasive field, this would be hard to achieve from small incision. Nevertheless, the deairing maneuver during minimally invasive cardiac operation is also hard to complete because the surgeon is unable to complete the deairing maneuver directly. However, echocardiography was repeated before discharge and showed a gradual improvement of cardiac function. Unfortunately, lactation did not occur, so it was possible to prescribe normal heart failure medications including a beta-blocker, aldosterone antagonist, and warfarin. However, the patient could hold her child as much as she wished without any of the limitations that would have been from a sternal procedure which has made mother-child bonding much easier and hence more successful.

In conclusion, the minimally invasive approach for atrial myxoma removal as a concurrent procedure with a mitral valve repair is feasible and safe with good cosmetic outcomes. Moreover, in a postpartum patient, this approach could avoid the numerous limitations resulting from a sternal incision and promotes excellent mother-child bonding during the crucial early period of a child's life.
